# Supplementary material for: An Evaluation of Ethograms Measuring Distinct Features of Enrichment Use by Captive Chimpanzees (Pan troglodytes)
Source: Animals (Basel). 2022 Aug 10;12(16):2029. doi: 10.3390/ani12162029 (PMC9404423; doi:10.3390/ani12162029)
Supplement: Supplementary file 1 [file animals-12-02029-s001.zip › animals-1783401-supplementary.pdf]

Table S1: Enrichment object ethogram. Duration of use measured for all enrichment objects.[a]

Object	Definition
Forage	Individual visibly touches food or touches objects in other categories[b] in order to obtain food from them at any point in the video. May include produce, bamboo, flowers, toys containing food, plates with food, cups with drink, chow (only when obtained from object), pools with food or water, bags with food, snow (only when indoors for enrichment purposes), object containing snow (e.g., bucket), hay bags, or straw containing food. Does not include chow by itself (without being obtained from object); plants naturally found naturally in outdoor enclosure such as weeds, sticks, and grass; snow when used outdoors; and any items that do not visibly contain food such as Kong toys, plates, cups.
Toys	Individual visibly touches smaller objects that allow utilization of fine motor skills and do not contain food or drink. May include balls, clothing, paper braids, pipes, puzzle devices, hammers and other tools, cleaning utensils, sandbox with sand, dolls, soap bubbles, plates, cups, straws, or paper bags.<br>Does not include boots worn by caregiver, nesting materials, sticks, mulch, plants found naturally in outdoor enclosure, and items containing food or drink.
Structural	Individual visibly touches enclosure structures and larger objects that allow perching, sitting, or utilization of gross motor skills such as climbing. May include barrels, pools, scooters, tires, cardboard boxes, ropes, bridges, firehoses, ladders, artificial trees, stools, tables, tunnels, or platforms. Does not include window lofts, logs, stumps, pools containing food, and unavoidable structures such as catwalks.[c]
Nesting	Individual touches flexible objects placed for the purpose of nesting. May include blankets, hammocks, cardboard paper, butcher paper, or straw. Does not include objects when food is foraged from within them or paper when used with art objects.
Technology	Individual examines or visibly touches digital devices, with devices remaining visible. May include iPad, digital camera, cell phone, or GoPro. Does not include any digital device that is not visible.
Art	Individual visibly touches items that allow opportunities for creative expression. May include crayons, paint, markers, or paper. Does not include paper when no other art material is present.
Other	Individual visibly touches any object that does not fit into other categories such as books, magazines, or mirrors. Note object.

[a]Objects were coded if an individual had at least 6 sec of physical contact with the object, or 10 sec of examine for technology. Tool use with objects and being inside of an object such as a box counted as physical contact.
[b]When an individual obtained food from within or on top of object or structure, both contact with enrichment object and contact with food were coded under the same duration instead of having coded food and object separately.
[c]Objects coded as structures had to be optional and avoidable. For example, a platform that was visibly part of the path from the indoors to the outdoors would have been unavoidable and, therefore, did not count as a structure. Other parts of structures, such as support beams, had to be visible so that it was clear to the observer that it was a structure and was avoidable.

Table S2: Enrichment manipulation behavior ethogram. Behaviors marked with an asterisk were treated as states rather than events and measured in seconds rather than frequencies.

Behavior	Definition
Carry	Individual drags object or moves with object in mouth, hands, or other body part more than two arms lengths from starting position without wearing object. Note whether moving toward or away from other individuals, if applicable.
Examine	Individual's gaze is focused on or head is facing towards digital technology for at least 10 seconds without breaking gaze, moving head away from, or manipulating object.
Oral	Individual brings food or nonfood enrichment object to its mouth and may bite, chew, kiss, suck, or lick object with object visible and without carrying object in mouth.[a]
Play on	Individual brachiates, swings, hangs, or climbs on enrichment.
Vocalize	Individual makes any sound with its vocal cords such as a hoot, laugh, screech, whimper, or grunt with mouth visibly moving and while in contact with object.[b]
Active tactile	Individual moves or manipulates object with body part, either with object moving or body part moving against object.[c]
Tool	Individual either a) uses a non-enrichment object to touch an enrichment object, b) uses an enrichment object to touch a non-enrichment object, or c) uses an enrichment object to touch another enrichment object. Tool may be used to clean, groom, or obtain food from object.[d]
Wear	Individual covers body part with object such as a blanket, clothing, box, or bag
Nest*	Individual may surround itself with objects, pull objects in close to itself, or flatten or otherwise manipulate objects to form a nest around its body
Rest*	Individual lies down while retaining physical contact with object but does not move body, move any object, or interact with others (except for retaining physical contact with others).[e]
Out of view*	Individual or manipulation behaviors with object are not visible before or after being seen manipulating object
Other	Individual engages in enrichment use behavior not previously listed. Note behaviors.

[a]New event began when object was taken out of or away from mouth and then brought back to mouth.
[b]If an individual vocalized more than once within 5 sec, it counted as one event in order to minimize potentially repetitive vocalizations.
[c]Did not include nest, tool, rest, or wear behaviors; bringing object to mouth; removing from mouth; or touching object while not moving object or body.
[d]If both objects were enrichment objects, tool use event was coded for each object. Physical contact was coded for each object that was an enrichment item on enrichment type ethogram; using a tool to manipulate an enrichment object counted as "physical contact" with said enrichment object.
[e]Individual may have been sleeping, close to falling asleep, or otherwise not moving.

Table S3: Social context of enrichment use ethogram. Durations measured for all social contexts.

Context	Definition
Solitary Use	Individual uses object alone without interacting with other chimpanzees, with no other chimpanzees interacting with object, without being in proximity (less than two arms' length) to other chimpanzees, and without engaging in affiliative use with staff.
Affiliative Use	Individual uses object while touching staff, uses object with staff touching object, gives object to staff, or plays with staff while touching object. Also includes playing or interacting with other chimpanzees while touching object or using object together. Individuals may exhibit play, grooming, or laughing while interacting. Note individual(s) involved.[a]
Proximate Use	Individual uses object with other chimpanzees in proximity (less than two arms' length) and without other chimpanzees interacting with the object or individual. Note individual(s) involved
Aggressive Use	Individual either a) directs aggression[b] towards conspecific with object or while touching object with individual screaming, conspecific screaming, or conspecific visibly fearful, or b) displays[c] with object in the presence of conspecifics while visibly hooting loudly or screaming. Note individuals(s) who are the target of the aggression, if any.
Submissive Use	Individual may flee or cower while touching object when conspecific approaches and may drop object while fleeing from conspecific without engaging in play behaviors. Note individual(s) involved

[a]Did not include staff giving food to individual.
[b]Examples included hitting or charging at conspecific while touching object or throwing, pushing, or kicking object towards conspecific.
[c]Displaying included either dramatically making loud noises with object or revealing strength with object, such as intensely slapping or beating object or picking up object and throwing it.

Table S4. Distribution of enrichment use occurrences by year and individual chimpanzee in the video archive. Numbers in parentheses are the number of videos for each chimpanzee included in the coded sample.

Year	Total Occurrences	Honey B	Mave	Willy B	Annie	Burrito	Foxie	Jamie	Jody	Missy	Negra	Multiple Chimpanzees
2021	127	26(6)	8(2)	13(3)	3(1)	43(9)	14(3)	9(2)	6(2)	4(1)	1(1)	15(4)
2020	347	108(22)	24(5)	66(13)	18(4)	32(7)	16(4)	34(7)	24(5)	13(3)	12(3)	53(13)
2019	270	26(6)	11(3)	13(3)	26(6)	43(9)	13(3)	59(12)	25(5)	32(7)	22(5)	39(10)
2018	415	-	-	-	48(10)	66(13)	54(11)	108(22)	43(9)	47(10)	49(10)	66(17)
2017	281	-	-	-	26(6)	42(9)	40(8)	105(21)	24(5)	21(5)	23(5)	33(9)
2016	264	-	-	-	21(5)	45(9)	51(11)	79(16)	22(5)	30(6)	16(4)	32(8)
2015	179	-	-	-	8(2)	41(9)	44(9)	51(11)	13(3)	5(1)	17(4)	25(7)
2014	210	-	-	-	15(3)	31(7)	49(10)	34(7)	29(6)	29(6)	23(5)	38(10)
2013	208	-	-	-	20(4)	37(8)	26(6)	58(12)	23(5)	25(5)	19(4)	23(6)
2011	238	-	-	-	18(4)	30(6)	72(15)	69(14)	20(4)	18(4)	11(3)	44(11)
